# Factors associated with refusal of preventive therapy after initial willingness to accept treatment among college students with latent tuberculosis infection in Shandong, China

**DOI:** 10.1186/s12879-023-08005-5

**Published:** 2023-01-20

**Authors:** Yemin Yuan, Jin Jin, Xiuli Bi, Hong Geng, Shixue Li, Chengchao Zhou

**Affiliations:** 1 Centre for Health Management and Policy Research, School of Public Health, Cheeloo College of Medicine, Shandong University, 44 Wenhuaxi Road, Jinan, 250012 Shandong China; 2 Department of Epidemiology, School of Public Health, Cheeloo College of Medicine, Shandong University, Jinan, 250012 China; 3 Public Health (Tuberculosis Prevention and Control) Centre, Shandong Public Health Clinical Center, Jinan, 250101 China; 4 NHC Key Laboratory of Health Economics and Policy Research, Shandong University, Jinan, 250012 China

**Keywords:** Latent tuberculosis infection, Tuberculosis, College students, Preventive therapy, Initial willingness, Refusal

## Abstract

**Background:**

Preventive therapy of latent tuberculosis infection (LTBI) is an important component of tuberculosis (TB) control. Research on acceptance of TB preventive therapy (TPT) is an important topic. Current studies focus on acceptability and compliance. However, it is unclear whether LTBI patients will start TPT after accepting treatment. The study assessed the factors associated with TPT refusal after initial willingness to accept treatment.

**Methods:**

Data were derived from a baseline survey of prospective study of LTBI treatment among college students in Shandong Province, China. A total of 723 students initially willing to accept TPT were included in the analysis. Stepwise logistic regression was used to explore the individual- and family-level characteristic variables that factors associated with TPT refusal after initial willingness to accept treatment.

**Results:**

Of the 723 LTBI college students who initially had acceptance willingness, 436 (60.3%) finally refused TPT. At the individual level, non-medical students were more likely to refuse TPT [odds ratio (OR) = 4.87, 95% confidence interval (CI): 3.10–7.67)], as were students with moderate physical activity (OR = 1.45, 95% CI: 1.04–2.04). Students with boarding experience (OR = 0.49, 95% CI: 0.31–0.78) and a high level of knowledge about TB (OR = 0.97, 95% CI: 0.95–0.99) were less likely to refuse TPT. At the family level, those with high father’s educational level (OR = 1.50, 95% CI: 1.07–2.10) or high household income (OR = 1.80, 95% CI: 1.20–2.71) were more likely to refuse TPT after initially accepting treatment.

**Conclusions:**

Factors associated with TPT refusal after initial willingness to accept treatment, such as personal (type of students, physical activity, boarding experiences, knowledge of TB) and family characteristics (father’s education level, household income) among college student with LTBI, might help identify persons for whom tailored interventions could improve the start of LTBI treatment.

**Supplementary Information:**

The online version contains supplementary material available at 10.1186/s12879-023-08005-5.

## Background

Tuberculosis (TB) is second only to COVID-19 as a leading cause of death from a single infectious agent, with 1.6 million deaths officially classified as caused by TB globally in 2021 [1]. TB is caused by the bacillus *Mycobacterium tuberculosis* (MTB). About a quarter of the world’s population is infected with MTB, and about 5–10% of those infected develop active TB in their lifetime [2, 3], becoming a new source of TB infection. Latent tuberculosis infection (LTBI) is a state of persistent immune response to stimulation by MTB antigens with no evidence of clinically manifest active TB [4]. China has the highest burden of LTBI, with nearly 350 million people infected with MTB [5]. TB preventive therapy (TPT) is an important and effective component of TB control.

An increasing number of studies have focused on the willingness to accept TPT. Previous studies in immigrants have determined concerns of stigma and reputational risk as relevant factors in willingness of TPT [6, 7]. Willingness to accept TPT was associated with the level of TB knowledge, the degree of exposure to patients with TB and family’s economic level among TB close contacts [8, 9]. In addition, predictors of willingness to accept TPT included personal beliefs, clinic accessibility, acculturation, concern about medication side effects, and level of LTBI knowledge [10–12]. In recent years, research on the acceptance of TPT has slowly transitioned from willingness to behavior. Studies found that lack of TPT readiness and substance use were barriers to treatment behavior, while LTBI and TB health education and familiarity with the homeless population were facilitators among homeless population [13]. Geographic and occupational were important influencing factors for starting and completing TPT [14]. The acceptability and adherence to TPT in HIV-infected patients were related to age [15]. Notably, losses before starting treatment accounted for greater net reduction of the public health benefits of LTBI management than did patient non-adherence with treatment once started [16]. However, whether LTBI patients will start TPT after accepting treatment has not received much attention. Little is known about factors associated with TPT refusal after initial willingness to accept treatment.

The incidence of TB is relatively low in most of China recently [17]. However, some TB outbreaks among college students were reported, drawing public concern. In China, the reported incidence of TB among students accounted for about 1/3 of that of the entire population, mainly high school and college students. The highest proportion was in the 18-year age group [18]. Evidence suggests that the incidence of TB in colleges was higher than in high schools in China [19]. An TB student has a large number of close contacts, which increases the prevalence of LTBI [20]. A recent systematic review and meta-analysis found that the prevalence of a positive tuberculin skin test (TST) was 20% (95% CI: 17–23%) among college students [21], higher than the previous LTBI prevalence [measured by the TST, 12% (95% CI: 10–13%)] among medical and nursing students in low- and middle-income countries [22]. Diagnosis and treatment of LTBI for college students could be highly beneficial to prevent TB reactivation. Previous studies have shown that TPT can achieve 60–90% protection and decrease the risk of active TB [23, 24]. Currently, the government has been increasingly concerned about TB control in schools. Management of active TB and LTBI are equally important. TPT has been piloted in some colleges in China [8], but reports on the factors associated with TPT refusal after initial willingness to accept treatment among college students with LTBI remain limited. Besides, intervention of LTBI of certain population requires optimization, as little is reported in this field.

In this study we aimed to examine factors that may influence TPT refusal after initially accepting treatment among college students with LTBI. The results of this analysis suggest strategies for developing TPT management in Chinese colleges.

## Methods

### Data source and sample

**Fig. 1:**
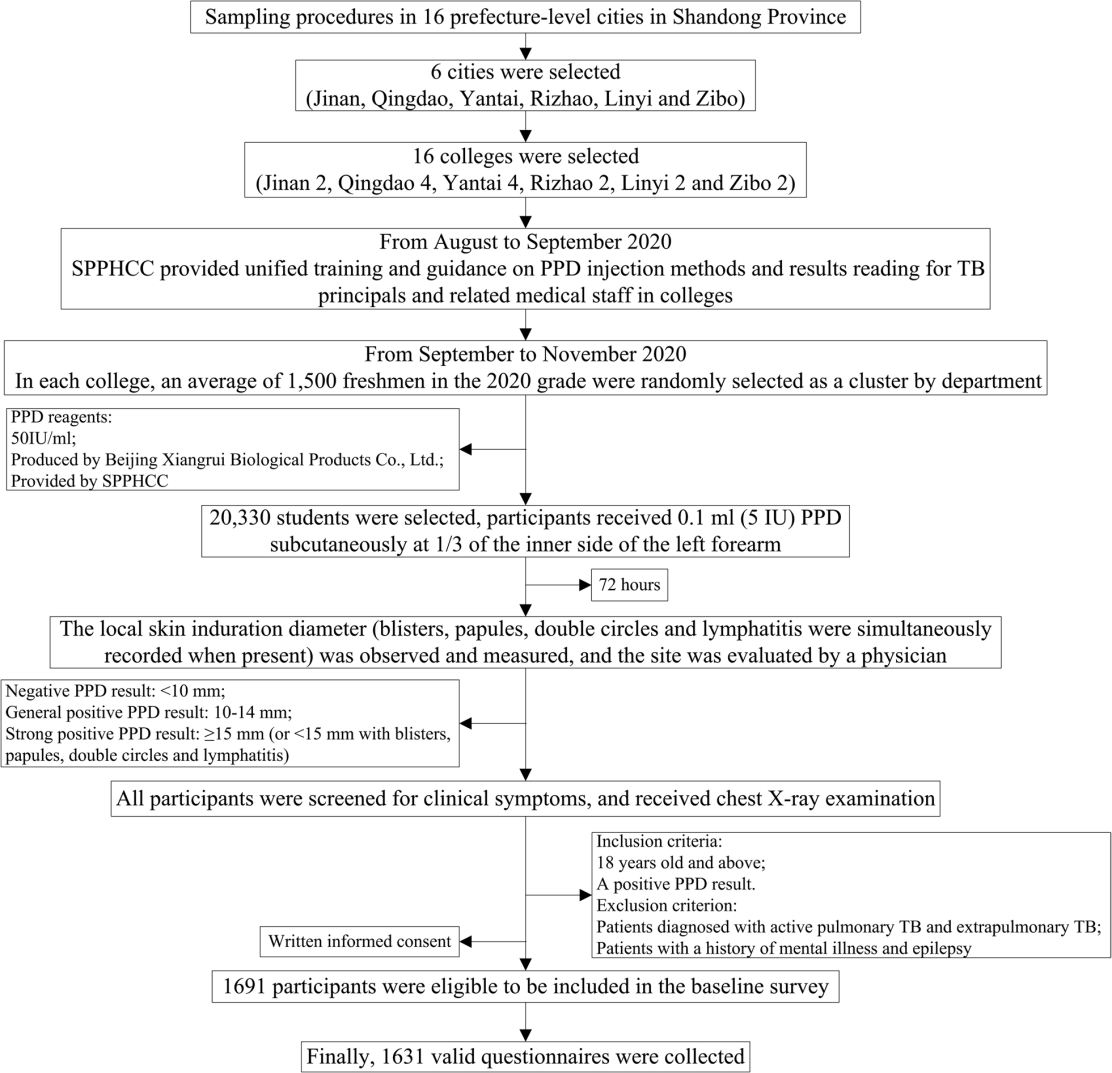
Flow chart of the sampling in this study. *SPPHCC* Shandong Provincial Public Health Clinical Center, *PPD* purified protein derivative, *TB* tuberculosis, *LTBI* latent tuberculosis infection. Induration diameter = (transverse diameter + longitudinal diameter)/2 (mm)

This study used the data from a baseline survey of prospective study of LTBI treatment among college students in Shandong, which was conducted from July to November 2020 in Shandong province, China. Specific procedure is shown in Fig. 1. A cluster random sampling method was adopted to select participants. Finally, 1691 respondents were instructed to fill in the electronic questionnaire by trained postgraduate students from the authors’ institute. All interviewers received adequate training to ensure the reliability of the survey. A total of 1631 valid questionnaires were collected. The distribution of participants across the colleges and cities was showed in Additional file 1.

Participants in this study were filtered by a question: “Are you willing to accept treatment of latent tuberculosis infection?” Those samples with the initial answer “Yes” were included in this study. When the medical staff asked the participants about their willingness, the students were given information about the meaning of the test, benefits of TPT, and potential side effects. The initial willingness was asked on the day the PPD results were checked, and the day before taking the medication (about one month and a half later) to determine whether to start treatment. Among 1631 participants, 723 who had initial willingness to accept TPT were included in the study.

### Dependent variable

The dependent variable was dichotomous. It was divided into “TPT acceptance after initial willingness to accept treatment” and “TPT refusal after initial willingness to accept treatment”.

### Independent variables

Independent variables were a series of variables that might affect whether respondents finally accept TPT based on literature reviews, including individual and family level variables. Variables at the individual level were age (continuous), gender (male, female), type of students (medical students, non-medical students), smoking (no, yes), current drinking (no, yes), physical activity (high, moderate, low), exposure history of TB (no, yes, unknown), boarding experiences (no, yes), and knowledge of TB (continuous). Physical activity was measured by the Chinese version of International Physical Activity Questionnaire Short Form (IPAQ-S) [25]. Boarding experiences means boarding at a school for at least one of the four stages of education (pre-school, primary education, primary secondary education and higher secondary education). Knowledge of TB consists of 17 questions that have been validated [26]. TB knowledge mainly covers the routes of TB transmission, symptoms, prevention, treatment and national policies. Details of TB knowledge are provided in Additional file 2. The score ranges from 0 to 45. The higher the score, the higher level of TB knowledge. At the family level, variables included residence (rural, urban), parents’ educational level (primary school or below, junior high school, high school or above), and household income in 2019 [quartile 1 (≤ 5800$), quartile 2 (> 5800$ and ≤ 10,150$), quartile 3 (> 10,150$ & ≤ 14,500$), quartile 4 (> 14,500$]. The average exchange rate in 2019 is 1￥ = 6.8967$.

### Statistical analysis

Data were analyzed using Stata 14.2 (Stata Corp, College Station, TX, USA). Descriptive statistics were used to describe baseline characteristics of participants with mean (standard deviation) for continuous variables and frequency (percentage) for categorical variables. Pearson’s chi-square test for categorical variables and Student’s t test for continuous variables were used to compare the characteristics of whether LTBI students refused TPT after initial willingness to accept treatment. The characteristics with *p* < 0.1 in the result of χ^2^ /t test were included in the logistic regression model. A stepwise selection method was used to select variables associated with TPT refusal after initially accepting treatment (the criteria for selection and elimination of variables were *p* < 0.05 and *p* < 0.10, respectively). We calculated the variance inflation factor (VIF) to check multicollinearity. VIF > 10 indicates severe multicollinearity. The odds ratio (OR) and 95% confidence interval (CI) of the variables were reported. The significance level was set as 2-sided *p* < 0.05.

## Results

Univariate analysis of TPT refusal after initial willingness to accept treatment is displayed in Table 1. Among 723 participants who were initially willing to accept TPT, the majority of them were non-medical students, and most of their parents were junior high school diplomas. They rarely smoke and drink. More than two-thirds had boarding experiences. The level of TB knowledge was generally low. 436 (60.3%) participants finally refused TPT. They were more likely to be non-medical students, have no boarding experiences, have a low level of TB knowledge, and have a high level of household income.

**Table 1:** Univariate analysis of TPT refusal after initial willingness to accept treatment among college students with LTBI

Variable	Total, n (%)	TPT refusal after initial willingness to accept treatment		^2^ / *t*	*p* values
		No	Yes		
Total	723(100.0)	287(39.7)	436(60.3)		
Age (years), mean ± SD	18.5 ± 0.8	18.5 ± 0.6	18.6 ± 0.8	− 1.751	0.080
Gender				3.163	0.075
Male	362(50.1)	132(46.0)	230(52.7)		
Female	361(49.9)	155(54.0)	206(47.3)		
Type of students				52.657	< 0.001
Medical students	112(15.5)	79(27.5)	33(7.6)		
Non-medical students	611(84.5)	208(72.5)	403(92.4)		
Smoking				0.160	0.689
No	644(89.0)	254(88.5)	390(89.5)		
Yes	79(11.0)	33(11.5)	46(10.5)		
Current drinking				0.013	0.910
No	540(74.7)	215(74.9)	325(74.5)		
Yes	183(25.3)	72(25.1)	111(25.5)		
Physical activity				2.797	0.247
Low	240(33.2)	102(35.5)	138(31.6)		
Moderate	258(35.7)	92(32.1)	166(38.1)		
High	225(31.1)	93(32.4)	132(30.3)		
Exposure history of TB				2.271	0.321
No	478(66.1)	196(68.3)	282(64.7)		
Yes	38(5.3)	11(3.8)	27(6.2)		
Unknown	207(28.6)	80(27.9)	127(29.1)		
Boarding experiences				8.464	0.004
No	116(16.0)	32(11.1)	84(19.3)		
Yes	607(84.0)	255(88.9)	352(80.7)		
Knowledge of TB (scores), mean ± SD	19.4 ± 7.5	20.1 ± 7.4	18.8 ± 7.5	2.356	0.019
Residence				1.934	0.164
Rural	375(51.9)	158(55.1)	217(49.8)		
Urban	348(48.1)	129(44.9)	219(50.0)		
Father’s educational level				6.341	0.042
Primary school or below	144(19.9)	58(20.2)	86(19.7)		
Junior high school	327(45.2)	144(50.2)	183(42.0)		
High school or above	252(34.9)	85(29.6)	167(38.3)		
Mother’s educational level				3.343	0.188
Primary school or below	230(31.8)	94(32.7)	136(31.2)		
Junior high school	303(41.9)	128(44.6)	175(40.1)		
High school or above	190(26.3)	65(22.7)	125(28.7)		
Household income				8.767	0.033
Quartile 1	190(26.3)	89(31.0)	101(23.2)		
Quartile 2	194(26.8)	75(26.1)	119(27.2)		
Quartile 3	166(23.0)	68(23.7)	98(22.5)		
Quartile 4	173(23.9)	55(19.2)	118(27.1)		

*TPT* tuberculosis preventive therapy, *LTBI* latent tuberculosis infection, *TB* tuberculosis, *SD* standard deviation

Table 2 shows the results of binary stepwise regression analysis. From personal factors, Non-medical students were more likely than medical students to refuse TPT after initial willingness to accept treatment (OR = 4.87, 95% CI: 3.10–7.67, *p* < 0.001). Participants with moderate physical activity were more likely to refuse TPT than those with low physical activity (OR = 1.45, 95% CI: 1.04–2.04, *p* = 0.031). Participants who had boarding experiences (OR = 0.49, 95% CI: 0.31–0.78, *p* = 0.003) and knowledge of TB (OR = 0.97, 95% CI: 0.95–0.99, *p* = 0.004) were less likely to refuse TPT after initially accepting treatment. From family factors, participants whose fathers had a high school education or higher were more likely to end up refusing TPT (OR = 1.50, 95% CI: 1.07–2.10, *p* = 0.018). Compared with participants with the lowest household income, participants with the highest household income were more likely to refuse TPT after initial willingness to accept treatment (OR = 1.80, 95% CI: 1.20–2.71, *p* = 0.005). The VIFs of independent variables in the model were all less than 1.5, indicating that there was no multicollinearity between independent variables.

**Table 2:** Binary stepwise logistic regression analysis of factors associated with TPT refusal after initial willingness to accept treatment among college students with LTBI

Variable	Model		
	OR	95% CI	*p* values
Age	1.24	0.99–1.55	0.060
Type of students			
Medical students	1.00		
Non-medical students	4.87	3.10–7.67	< 0.001
Physical activity			
Low	1.00		
Moderate	1.45	1.04–2.04	0.031
High	1.03	0.69–1.54	0.868
Boarding experiences			
No	1.00		
Yes	0.49	0.31–0.78	0.003
Knowledge of TB	0.97	0.95–0.99	0.004
Father’s level of education			
Primary school or below	1.00		
Junior high school	0.80	0.51–1.23	0.303
High school or above	1.50	1.07–2.10	0.018
Household income			
Quartile 1	1.00		
Quartile 2	1.38	0.95–2.02	0.095
Quartile 3	1.05	0.66–1.67	0.806
Quartile 4	1.80	1.20–2.71	0.005

*TPT* tuberculosis preventive therapy, *LTBI* latent tuberculosis infection, *TB* tuberculosis, *OR* odds ratio, *CI* confidence interval

## Discussion

Our analysis of factors associated with TPT refusal after initially accepting treatment. The most important finding was that among 723 (44.3%) college students who initially accepted treatment, 436 (60.3%) refused TPT after 1.5 months. However, no evidence of TPT refusal after initial willingness to accept treatment has been reported before this study. Our findings underscore that the factors associated with TPT refusal after initially accepting treatment appear to differ in some respects.

First, it is noteworthy that non-medical college students were associated with an increased likelihood of TPT refusal compared with medical students. One possible explanation is that non-medical students lack important TB knowledge and have misconceptions about TB [26, 27]. Despite we had a TB-related education (diagnosis, common symptoms, transmission routes, hazards and ways to seek medical treatment) for college students at baseline, it might not have much effect on non-medical students in the end. Misunderstanding is another possible explanation. The observed critical misconceptions about diseases could be a major barrier to appropriate behavioural choices [28, 29]. Perhaps non-medical students seldom have a comprehensive understanding of TB and LTBI. Colleges should popularize students about the difference between TB and LTBI to eliminate misconceptions. Schools should also enable non-medical students to master certain health science knowledge.

Second, LTBI students with moderate physical activity were more likely to end up with TPT refusal compared with LTBI students with low physical activity. This finding is inconsistent with previous results of older adults with diabetes. There was no statistical association between physical activity and attitudes towards TPT [12]. The difference in findings between students and older adults could be attributable to participation rates in physical activity. Participation in physical activity remains low among older adults [30], which may make it difficult to detect a relationship between physical activity and TPT acceptance. In fact, physical activity is essential to health among the youth. Only about one-third of Chinese college students achieved the National Fitness Program’s recommendation for moderate physical activity [31]. Students who more physically active had better self-perceived health [32], and they did not think they had any health problems [33]. Colleges can help students develop good living habits, pay attention to work and rest, pay attention to the development of physical education. Colleges can also further improve the form and content of physical education and provide students with colorful physical education.

Our study reveals that LTBI college students with boarding experiences and a high level of TB knowledge were much less likely to refuse TPT after initial willingness to accept treatment. Boarding is a common phenomenon for middle or high school students in China. A previous report also revealed boarding schools were more subject to a higher risk of TB outbreaks, where students often cluster under relatively overcrowded conditions, compared to other school settings [34]. This result indicates that former boarding students may have a history of TB exposure and be afraid that they will develop TB. In addition, students with lower scores of TB knowledge were less likely to accept TPT [8]. In our study, these students with a lower level of TB knowledge were also more likely to refuse TPT after initially accepting treatment. College should integrate the health education of TB and LTBI knowledge into the overall planning of teaching and health prevention. Various forms of publicity and education activities should be carried out regularly and continuously to improve awareness of TB among college students with LTBI.

This study also found that LTBI college students whose fathers had a high school education or above were more likely to end up refusing TPT. This finding is inconsistent with previous studies. A study in a vocational school in Guangzhou, southern China indicated students whose parents had a low education level were prone to be non-adherent with TPT [35]. This discordant finding suggests that parents’ educational level can have different impacts on whether students accept TPT or not in different areas or stages. Our findings might be responsible that parents with a high level of education have some knowledge about TB, but they don’t know about LTBI. Parents may be more concerned about prolonged treatment duration, unobservable long-term effects, and possible side effects of TPT. This makes it difficult for them to allow TPT for their children. However, our result might also highlight the importance of incorporating the latest evidence of the effectiveness of treatment programmes into LTBI health education. Colleges should also strengthen communication with students’ parents.

In our study, TPT refusal after initially accepting treatment among LTBI college students with high household income is similar to previous studies. A study conducted in the United States and Canada for the general population found that the percentage accepting TPT declined as income increased [10]. A study of TB close contacts in China also showed that people with the highest household income per capita were more likely to refuse preventive medication than those with the lowest income [9]. Possible explanations are that, in this survey, free medications and inspections have less incentive effect on LTBI college students with better family financial conditions than those with poorer family financial conditions. LTBI college students from low-income families are afraid that if they develop TB, they will increase the financial burden on the family.

The findings in the current study imply that the management of LTBI in colleges needs to pay attention to those students who are non-medical students, moderate physical activity, no boarding experiences, a lower level of TB knowledge, high household income, and high father’s educational level, because these students are prone to end up refusing TPT. Colleges need to strengthen the publicity and education of TB and LTBI knowledge and enhance the knowledge of LTBI, so as to improve the TPT acceptance. In addition, parents’ awareness of TB and LTBI should be raised. Proper management of LTBI among college students is crucial to achieve public TB control. One limitation of this study was that our study was conducted in Shandong province, and the conclusions cannot be generalized due to the lack of other regional or national surveys. We do not have information on why the students refused TPT before treatment, which will be further explored in future studies. We also suggest that future studies can investigate the effect of the duration from LTBI diagnosis to treatment on acceptance.

In conclusion, this study prospectively provides important new information on factors associated with TPT refusal after initial willingness to accept treatment among LTBI college students. Type of students, physical activity, boarding experiences, knowledge of TB, father’s level of education, and household income influence the final acceptance of TPT. These findings have important implications for LTBI management. The management of LTBI college students should pay attention to these factors to improve students’ TPT acceptance.

## Supplementary Information

**Additional file 1. Table S1.** The distribution of participants across the colleges and cities.

**Additional file 2.** Knowledge of tuberculosis.

## Data Availability

The datasets used and analyzed during the current study are available from the corresponding author on reasonable request.

## Abbreviations

TB: Tuberculosis
MTB: *Mycobacterium tuberculosis*
LTBI: Latent tuberculosis infection
TPT: Tuberculosis preventive therapy
TST: Tuberculin skin test
IPAQ-S: International Physical Activity Questionnaire Short Form
VIF: Variance inflation factor
