# Supplementary material for: Factors associated with refusal of preventive therapy after initial willingness to accept treatment among college students with latent tuberculosis infection in Shandong, China
Source: BMC Infect Dis. 2023 Jan 20;23:38. doi: 10.1186/s12879-023-08005-5 (PMC9857917; doi:10.1186/s12879-023-08005-5)
Supplement: Supplementary file 1 — Additional file 1. Table S1. The distribution of participants across the colleges and cities. [file 12879_2023_8005_MOESM1_ESM.doc]

**Supplementary file 1: Supplemental Table 1**

Table S1 The distribution of participants across the colleges and cities

| Variable | n | % |
| --- | --- | --- |
| Total | 1631 | 100.0 |
| City |  |  |
| City 1 | 177 | 10.9 |
| City 2 | 453 | 27.8 |
| City 3 | 167 | 10.2 |
| City 4 | 182 | 11.2 |
| City 5 | 327 | 20.0 |
| City 6 | 325 | 19.9 |
| College |  |  |
| College 1 | 100 | 6.1 |
| College 2 | 77 | 4.7 |
| College 3 | 232 | 14.2 |
| College 4 | 221 | 13.6 |
| College 5 | 84 | 5.2 |
| College 6 | 83 | 5.1 |
| College 7 | 67 | 4.1 |
| College 8 | 115 | 7.0 |
| College 9 | 116 | 7.1 |
| College 10 | 54 | 3.3 |
| College 11 | 35 | 2.1 |
| College 12 | 122 | 7.5 |
| College 13 | 27 | 1.7 |
| College 14 | 140 | 8.6 |
| College 15 | 76 | 4.7 |
| College 16 | 82 | 5.0 |
