# Supplementary material for: Factors associated with refusal of preventive therapy after initial willingness to accept treatment among college students with latent tuberculosis infection in Shandong, China
Source: BMC Infect Dis. 2023 Jan 20;23:38. doi: 10.1186/s12879-023-08005-5 (PMC9857917; doi:10.1186/s12879-023-08005-5)
Supplement: Supplementary file 2 — Additional file 2. Knowledge of tuberculosis. [file 12879_2023_8005_MOESM2_ESM.doc]

**Supplementary file 2: Knowledge of tuberculosis**

Knowledge of tuberculosis consists of the following 17 questions. The scoring rules for each question are marked at the end of the question.

1. Have you heard about TB? (Choose option 2 and score one point.)

1=No 2=Yes

1. Is TB communicable disease? (Choose option 2 and score one point.)

1=No 2=Yes 3=I have no idea

1. What organs of human body can be affected by TB? (Please check all that are mentioned.) (Choose option 1/2/3/4/5 to score one point respectively. One point if option 6 is reasonable.)

1=Bones 2=Kidney 3=Uterus 4=Abdomen 5=Lungs

6=Others, Specify_____ 7=I have no Idea

1. How can a person infected with TB transmit it to another person? (Choose option 2 and score one point.)

1=Through touching items in public places

2=Through the air when the infected Person coughs or sneezes

3=Through eating from the same plate 4=Through handshakes

5=I have no Idea

1. Which of the following are signs or symptoms of TB affecting the lungs? (Please check all that are mentioned.) (Choose option 1/2/3/4/5/6/7/8 to score one point respectively. One point if option 9 is reasonable.)

1=Fever 2=Hemoptysis/Coughing up blood 3=Night sweats

4=Loss of appetite 5=Chest pain 6=Loss of weight 7=General weakness

8=Cough≥2weeks 9=Others, Specify_____ 10=I have no Idea

1. How can one prevent infection with TB? (Please check all that are mentioned.) (Choose option 1/2/3/6/8/9/10 to score one point respectively. One point if option 11 is reasonable.)

1=Covering mouth and nose when coughing or sneezing

2=Avoid sharing dishes

3=Washing hands after touching items in public places

4=Closing windows at home

5=Closing windows during public transportation (bus, taxi)

6=Through good nutrition 7=By prayer 8=By vaccination

9=By isolating TB patients 10=Avoid shaking hands

11=Others, Specify_____ 12=I have no Idea

1. Can TB be cured? (Choose option 2/3/4 to score one point respectively)

1=No 2=Yes, completely 3=Yes, Partially 4=Yes 5=I have no Idea

1. What is the best treatment for someone with TB? (Choose option 4 and score one point.)

1=Herbal Remedies 2=Homemade Remedies 3=Prayer

4=Specific drugs given at health facilities/Modern medicine 5=I have no Idea

1. How long is the TB treatment? ______months (If you don’t know, fill in “0”.) (Fill in 6 months or more to score one point )
2. Which of the following are risks if a person with TB does not take treatment? (Please check all that are mentioned.) (Choose option 1/2/3/5 to score one point respectively. One point if option 6 is reasonable.)

1=Infects others 2=Losses weight 3=Develops sever health problems

4=No effect 5=Death 6=Others, Specify_____ 7=I have no Idea

1. Which of the following are risks if a person with TB does not finish a full course of treatment? (Please check all that are mentioned.) (Choose option 1/2/3/5 to score one point respectively. One point if option 6 is reasonable.)

1=Drug resistance 2=Relapse 3=Inability to cure infection

4=No effect 5=Death 6=Others, Specify_____ 7=I have no Idea

1. Do you know if you have a continuous cough and expectoration for more than 3 weeks (or blood in your sputum) should you suspect tuberculosis? (Choose option 1 and score one point.)

1=Yes 2=No 3=I have no Idea

1. Where is the general establishment of professional institutions for tuberculosis prevention and treatment in China? (Choose option 1 and score one point.)

1=The disease prevention and control center (epidemic prevention station) of the county and above

2=Pulmonary department of general hospitals at and above the county

3=I have no Idea

1. Do you know that the country implements a reduction or exemption policy for the treatment of tuberculosis patients? (Choose option 1 and score one point.)

1=Yes 2=No 3=I have no Idea

1. Where do you think tuberculosis patients can be checked and treated for free? (Choose option 3 and score one point.)

1=Local township (street) health centers (infirmary)

2=General hospital at county level and above

3=Local disease control center (epidemic prevention station)

4=I have no Idea

1. Do you think that as long as you adhere to regular treatment, most patients can be cured? (Choose option 1 and score one point.)

1=Yes 2=No 3=I have no Idea

1. Do you know what kind of tuberculosis patients are contagious, which means they can infect other people? (Choose option 2 and score one point.)

1=Patients with cough and sputum

2=Patients whose sputum can be detected with Mycobacterium tuberculosis

3=All tuberculosis patients

4=I have no Idea
